# Psycho-Microbiology, a New Frontier for Probiotics: An Exploratory Overview

**DOI:** 10.3390/microorganisms10112141

**Published:** 2022-10-29

**Authors:** Alessandra Accettulli, Maria Rosaria Corbo, Milena Sinigaglia, Barbara Speranza, Daniela Campaniello, Angela Racioppo, Clelia Altieri, Antonio Bevilacqua

**Affiliations:** Department of Agriculture, Food, Natural Resources and Engineering (DAFNE), University of Foggia, 71122 Foggia, Italy

**Keywords:** trials, research paper, mental health, probiotics

## Abstract

Probiotics are gradually gaining importance in the field of psychiatry in the form of psychobiotics. Psychobiotics’ studies examine the existing relationship between gut microbiota and mental phenomena; the intake of certain strains of probiotics, such as *Bifidobacterium* and *Lactobacillus*, for example, allow the gut microbial system to be modified in order to provide benefits at the psychic, immune, hormonal, and mental levels. Those who suffer from forms of depression, anxiety disorders, chronic stress, low mood, but also people who do not suffer from such disorders, can therefore benefit from the use of psychobiotics. Thanks to probiotics, neurochemicals can in fact be produced within the gut microbiota and interact with receptors of the enteric nervous system that innervate the entire gastrointestinal tract. Once they enter the portal circulation, these substances go on to influence components of the nervous system and ultimately the brain, through what is called the gut–brain axis. This article proposes an exploratory overview of the proven effects of probiotics on brain activity and psycho-related diseases, focusing on clinical studies and measurable outcomes. The search was conducted using two different online tools: ClinicalTrials.gov and PubMed.

## 1. Probiotics, Gut Microbiota, and Mental Health: An Introduction

The term probiotic is used to designate microorganisms that can promote human health and well-being. According to the official definition, probiotics are live and viable microorganisms that, when administered frequently and in adequate amounts, benefit the health of the host [1]. They share the following requirements and common traits [2]:

- They are safe for use in humans;
- They are active and viable in the gut, and present in sufficient quantity to justify any beneficial effects;
- They have an active metabolism in the human intestine, and some strains could persist and multiply;
- They confer a demonstrated physiological benefit.

Probiotics must not carry acquired and/or transmissible antibiotic resistance; moreover, it is worth mentioning that their colonization in the intestine is only temporary in nature and ends a few days after discontinuation of their intake.

The clinical effects of probiotics depend on specific bacteria, which should be defined not only by genus and species, but also at the strain level [3]. Although there are a few clinical studies that directly compare the effects of various strains within a single species, the available evidence shows that some effects of probiotics are strain-specific, while others may be species-specific. In any case, the clinical effect of a probiotic microorganism should be attributed only to the strains that have been demonstrated to possess it. Conversely, when one strain is not eliciting a certain clinical effect, it does not mean that no other strain can produce it. Therefore, in order to be the subject of a consumer health claim, proper characterization must be carried out [3].

The term gut microbiota defines the set of living species that colonize the human gastrointestinal tract, which is considered one of the most densely populated ecosystems in the world with an estimated 100 trillion microorganisms including bacteria, yeasts, protozoa, and viruses. When these communities are in balance with each other, a condition called eubiosis is established [4]. This is particularly important because it allows the various components of the gut microbiota to be functionally effective, and above all to be synchronized both with each other and with the other components of the gut ecosystem. Under these conditions, the microbiota can perform several essential functions for the host, including developmental, immunological, nutritional, infection prevention, nutrient acquisition, and brain and nervous system functions [4]. Consequently, the role that a well-balanced microbiota plays in eubiosis is critical to the overall health of the body. When this balance is lacking, a condition called dysbiosis is established and can cause various diseases, metabolic disorders, and affect human life.

Gut microbiota changes throughout the course of life, providing each individual with a specific “fingerprint.” Indeed, it develops at birth, diversifies by increasing in composition and becomes stable between the third and seventh decade of life. It is then with aging that its microbial diversity tends to decrease, probably influenced by changes in digestion, nutrient absorption, and weakening of immune activity [5].

Studies have shown the presence of 10 predominant microbial phyla in the human gut microbiota. In particular, the phyla Firmicutes, Bacteroidetes, and Proteobacteria represent 90% of the total; the phyla Actinobacteria, Fusobacteria, and Verrucomicrobia account for the remaining 10% [5].

In this context, probiotics have a key role, namely to help maintain the health of the microbiota, but also to bring back into balance a microbiota attacked by pathogens or physical stresses [6].

Microbiota and the brain are capable of exerting a strong influence on each other through a bidirectional connection, called the gut–brain axis. They communicate through various pathways, including the immune system, tryptophan metabolism, vagus nerve, and enteric nervous system, involving microbial metabolites such as short-chain fatty acids, branched-chain amino acids, and peptidoglycans acting as neurotransmitters [7]. This connection not only serves to ensure the proper digestive pathway, but is involved in a multitude of physiological processes including satiety, food intake, regulation of glucose and fat metabolism, hormone secretion and sensitivity (especially to insulin), and bone metabolism. Moreover, thanks to gut microbiota, it is also possible to positively modulate cognitive functions, and it is becoming increasingly evident how important it is in fields studying the biological and physiological basis of psychiatric and neurodegenerative disorders [7].

Classic endocrine organs such as the pituitary, thyroid, pancreatic islets, adrenal glands, and gonads produce hormones that are transported by the blood to exert their effects on distant organs through specific receptors. However, it has become apparent not only that these organs produce hormones, but also that all organs communicate with each other through the secretion of specific hormones, exerting effects at a distance depending on the expression of the appropriate receptors [8].

The brain, following food intake, receives input from the gut, and determines physiological responses together with signals from other organs [8].

Some years ago, the term psychobiotics was introduced to define a new class of probiotics able to produce substances that can affect the gut–brain connection, improve mood, decrease anxiety and depression, and bring many other benefits. The term itself suggests the connection both with the psyche and with the world of probiotics; these microorganisms are in fact able to perform positive functions in the intestine and perpetrate their positive effect in the brain as well, communicating through the bidirectional intestine–brain axis [9].

Recent studies, mainly carried out on animal models, support the role of microbes as signaling components in the gut–brain axis, and it has been established that the vagus nerve is the main route for exerting the effects of gut microbiota on the central nervous system.

There are many probiotics that have shown a positive effect in this regard; for example, treatment with *Bifidobacterium* spp. can increase the amount of tryptophan, precursor of serotonin, the hormone of happiness. Some *Lactobacillus* and related genera species alter gamma-aminobutyric acid (GABA) metabolism and change brain GABA receptor expression and behavior. Synthesis and release of neurotransmitters from bacteria have been reported: *Lactobacillus* and *Bifidobacterium* species can produce GABA; *Saccharomyces* spp., *Escherichia,* and *Bacillus* can produce noradrenaline; *Streptococcus*, *Enterococcus* spp., *Escherichia*, and *Candida* can produce serotonin; *Bacillus* can produce dopamine; and *Lactobacillus* can produce acetylcholine [10]. 

Exploration of the gut microbiota has shown clear benefits on behavior and brain function, and may also aid in the treatment of depression [11]. Already in current practice, the treatment of anxiolytic and antidepressive-like behavior has been mediated with *Lacticaseibacillus rhamnosus* JB-1 oral therapy through the gut–brain axis. Moreover, from numerous studies it was found that a quantifiable set of microbial markers was consistently present in the feces of depressed subjects. These markers can also be used to determine the severity of disease progression.

However, despite the increased interest in psychobiotics, there are some controversial results; therefore, the aim of this paper is to search for proven effects of probiotics on brain activity and psycho-related diseases, with a focus only on clinical trials and measurable outputs. The search was conducted using two different online tools: ClinicalTrials.gov and PubMed.

## 2. ClinicalTrials.gov

**Figure 1:**
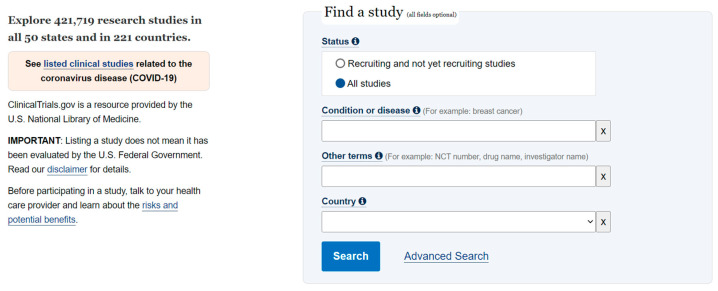
Dashboard of ClinicalTrials.gov.

Clinical Trials is a database of privately and publicly funded clinical studies conducted around the world; it is provided by the US National Library of Medicine [12]. Within the database (Figure 1), it is possible to search for a specific condition or disease by using certain keywords or countries. 

The term “probiotic” was used as a keyword. After conducting the search, the “By Topic” button was pressed in order to have the results divided by category. To reduce the number of studies, topics with at least 10 clinical trials were selected. Next, the clinical studies were entered into an Excel sheet, with the following details (Table 1):

- topic title;
- number of trials;
- trials with clinical results.

After this screening, attention was paid to topics connected with the gut–brain axis; thus, nine trials with results were found. The details (topic, title, kind of probiotic) are shown in Table 2.

A main drawback of this new research was the low number of studies with published results at the time of the research (only four), while other trials did not clearly report the results, or they showed a table without a statistical analysis.

From the evidence available on ClinicalTrials.gov, it could be concluded that probiotics:

(a) could exert an indirect positive effect on veterans affected by post-traumatic stress, due to a reduction in some inflammatory markers (C reactive protein) after the treatment with *Limosilactobacillus reuteri*;
(b) are associated with a low tendency to develop severe bowel difficulty in patients with schizophrenia after supplementation with *Lcb. rhamnosus* and *B. animalis*;
(c) are positively linked to a lower rate of rehospitalization in patients affected by mania, after treatment with *Lcb. rhamnosus* and *B. animalis*;
(d) determine a lower rate of self-reported anxiety after treatment with *Lcb paracasei*.

## 3. Search on PubMed

PubMed was used for a second search. Two filters were set: a temporal range of 5 years and a focus only on clinical trials, using the following keywords: mental health, anxiety, gut brain–axis, and probiotics. Thus, ten records were selected, briefly described in this section; these studies focused on the effects of probiotics through standardized questionnaires and surveys, reported in Table S1 (Supplementary Materials).

Tran et al. [17] conducted their assessments on college students (*n* = 86), healthy young adults aged 18 to 31 without severe psychological disturbances. They were asked to consume a daily mix of probiotics for 28 days; the mix was composed of multiple strains of *Bifidobacterium* spp., *Lactobacillus* spp., *Streptococcus salivarius* subsp. *thermophilus*, *Bacillus coagulans*, and *Lactococcus lactis*, with concentrations ranging from 1 × 10^10^ to 5 × 10^10^ CFU; before and after the trial, they completed: BAI (Beck Anxiety Inventory), a self-report tool, to evaluate the severity of anxious symptoms; ACQ-R (Anxiety Control QuestionnaireRevised), for the perceived level of control over anxiety-related events; PSWQ (Penn State Worry Questionnaire), to measure feelings of worry; NMR (Negative Mood Regulation), a questionnaire that assesses an individual’s perception of his or her ability to regulate negative mood; and PANAS (Positive Affect and Negative Affect Scales), to evaluate positive (PA) and negative (NA) affective states. A multiple regression approach on the results pointed out that probiotics reduced panic anxiety in 35.41% of cases. An important outcome of the research was that a ceiling effect of probiotics was found in some subjects; thus, the authors concluded that anxiety was reduced only in people with a high level of stress, with promising results in all volunteers in terms of worry, negative affect, mood regulation, and anxiety control.

Lew et al. [18] conducted a 12-week randomized, double-blind, placebo-controlled study on the effects of *Lactiplantibacillus plantarum* strain P8 (10 log CFU/day) in 103 stressed adults. Cortisol and cytokine levels in plasma were evaluated, and participants completed CogState tests to evaluate cognition and cognitive functions, as well as the PSS-10 questionnaire (Perceived Stress Scale) at 4-week intervals (4-8-12) on the perception of stress and questions relating to feelings and thoughts, and the DASS-42 questionnaire (Depression Anxiety and Stress Scale-42 items), a 42-item self-report scale designed to measure emotional states of depression, anxiety, and stress. The data obtained showed a reduction in some inflammatory cytokines, and enhanced memory and cognitive traits; this also depended on the individual’s sex as the effects were higher in men than in women due, as suggested by authors, to the higher tendency of externalization symptoms in men.

Probiotics were used in two studies to treat schizophrenia [19,20]. In the study of Okubo et al. [19] *B. breve* A-1 was administered to a group composed of 30 volunteer patients. In this open-label single-arm study, all participants first completed a questionnaire on demographics and on food frequency and then received *B. breve* A-1 strain (10^11^ CFU/day, two sachets per day) for 4 weeks followed by 4 weeks of observation. The syndrome scale was evaluated through the Positive and Negative Syndrome Scale (PANSS), blood test results, and fecal microbiome composition. Based on results, volunteers were divided into two groups: responders to probiotics and non-responders. Generally, probiotics determined a significant improvement in the Hospital Anxiety and Depression Scale (HADS) scores and the PANSS anxiety/depression scores after 4 weeks; moreover, a higher number of *Parabacteroides* in the feces and a reduction in symptoms were recorded in responders.

In the second case [20], a probiotic mix formed by *Lcb. rhamnosus* and *B. animalis* subsp. *lactis* was administered for 14 weeks with schizophrenic patients (*n* = 56) to evaluate the levels of antibodies active against *C. albicans* (saprophytic fungus in the gastrointestinal tract); these antibodies, according to some recent experimental evidence, seem to be correlated with the development of syndromes of a psychotic nature in patients suffering from schizophrenia. The experimental design was based on a longitudinal, double-blind, placebo-controlled study. Participants were required to complete the PANSS questionnaire at the beginning, and again every two weeks. Antibodies decreased by 43% in the volunteers taking the probiotic mix, and by only 3% in the subjects receiving the placebo.

In another clinical study (double-blind, with placebo), it was shown that *B. longum* 1714™ could significantly reduce cortisol production and stress responses in healthy subjects exposed to an acute stress factor. Volunteers (*n* = 40; aged 18–50) received the probiotic or placebo for four weeks. The effects on the responses to social stress were studied, induced by the “Cyberball game” and a standardized paradigm of social stress. Brain activity was measured using magnetoencephalography (MEG), and then the quality of life assessment questionnaire (SF-36) was performed. The outputs showed that the probiotic could alleviate mental fatigue, modulating the response to social stress at the same time through a controlling effect on the neural oscillations in several brain regions [21].

The effect of consuming soy fermented with *Lcp. plantarum* C29 (DW2009) was shown to improve the cognitive function of individuals with MCI (mild cognitive impairment). Patients with this disorder (*n* = 100; 55–85 years) received the probiotic or the placebo (800 mg/day) for 12 weeks. Computerized neurocognitive function tests (CNT) performed at baseline and at the end of the study to assess associations between changes in brain neurotrophic factor (BDNF) levels and cognitive performance for each group. Volunteers taking the probiotic showed improved cognitive functions in the attention domain, probably related to an increased level of serum brain-derived trophic factor [22].

Nishida et al. [23] studied the effect of *L. gasseri* CP2305 on the improvement of clinical symptoms in healthy young adults and in patients with irritable bowel. This research was a double-blind, placebo-controlled clinical study carried out from July 2017 to March 2018 on Japanese medical students (*n* = 60) preparing to sit the national medicine examination. Volunteers were asked to ingest tablets containing CP2305 or placebo once a day for 24 weeks, and were then submitted to electroencephalogram (EEG) to monitor brain activity during the night; the results showed that taking the probiotic significantly reduced anxiety and sleep disturbance compared to the placebo group, as demonstrated by the Spielberger State-Trait Anxiety Inventory and the Pittsburgh Sleep Quality Index. In addition, *L. gasseri* CP2305 reduced sleep latency and wake time after sleep onset, increased the delta power ratio in the first sleep cycle, and lowered salivary chromogranin A levels.

Next, the effect of probiotic consumption on cognitive patterns associated with depression was examined; the reference population consisted of patients with mild and severe depression [24]. This is a triple-blind randomized control and placebo control. Volunteers consumed Winclove’s Ecologic^®^ Barrier probiotic supplements (which consists of nine bacterial strains: *B. bifidum* W23, *B. lactis* W51, *B. lactis* W52, *L. acidophilus* W37, *Levilactobacillus brevis* W63, *Lcb. casei* W56, *Ligilactobacillus salivarius* W24, *L. lactis* W19, and *L. lactis* W58). All participants (*n* = 70) were randomly assigned a probiotic mix or placebo, which was consumed daily for eight weeks. All volunteers were asked to complete the following tools: M.I.N.I. (Mini-International Neuropsychiatric Interview), a semi-structured diagnostic rating scale; BDI-II (Beck Depression Inventory-II) a self-report tool that facilitates assessing the severity of depression in patients with a psychiatric diagnosis; DASS-21 (Depression Anxiety and Stress Scale-21 items); BAI (Beck Anxiety Inventory); LEIDS-R (Leiden Index of Depression Sensitivity-Revised); a post-evaluation questionnaire, a dietary questionnaire, and a customer satisfaction questionnaire were also carried out. The results were contradictory, as further investigations were probably necessary, but a significant difference was found between the probiotic and placebo groups, particularly in the mild–moderate depression severity subgroup on a measure of cognitive reactivity towards sad mood, which is a vulnerability marker of depression.

Kazemi et al. [25] evaluated the effect of probiotics on patients with major depressive disorder (MDD), with a double-blind experimental design using the Beck Depression Inventory (BDI) score, on the kynurenine–tryptophan ratio and on the tryptophan–branch chain ratio (BCAA).

Patients (*n* = 110; mean age, 36.5 years) were randomly assigned a probiotic (*L. helveticus* and *B. longum*), prebiotic (galacto-oligosaccharide), or placebo for eight weeks. Overall, eight weeks of probiotic supplements in subjects with MDD resulted in an improvement in the BDI score compared to placebo, while no significant effect of the prebiotic was recorded.

Instead, studies on *Lcb. rhamnosus* HN001 have shown a positive effect on maternal depression in the postpartum period [26]. It was a randomized, double-blind, placebo study conducted in New Zealand in a sample population of women (*n* = 423). They were recruited during gestation at 14–16 weeks, and received a placebo or HN001 from the day of enrolment up to 6 months post-partum while breastfeeding. They filled out a baseline questionnaire at the start of the study, and then a questionnaire on psychological well-being after childbirth. Mothers in the probiotic treatment group reported significantly lower depression scores than those in the placebo group.

A tentative overview of the most important outcomes and benefits of some species are in Table 3.

## 4. Conclusions

The outcomes of studies on the use of psychobiotics appear to be very promising; however, for some topics there are controversial details, while for other pieces of research the data are still too scarce to support a strong hypothesis.

Gut microbiota is not a silent organ, and the microbial communities that populate it are active participants in determining the well-being of the host, and also for mental health through the bidirectional pathway known as the gut–brain axis. Some strains or mixes could positively contribute to situations involving reduction in anxiety, sleep-related disorders, improvement of cognitive function, reduction in stress and thus cortisol levels, or reduction in mental fatigue.

However, there is a long way to go; some benefits are documented, while others are only postulated. Moreover, it is important to understand the exact mechanisms by which probiotics could act as psychobiotics.

**Figure 2:**
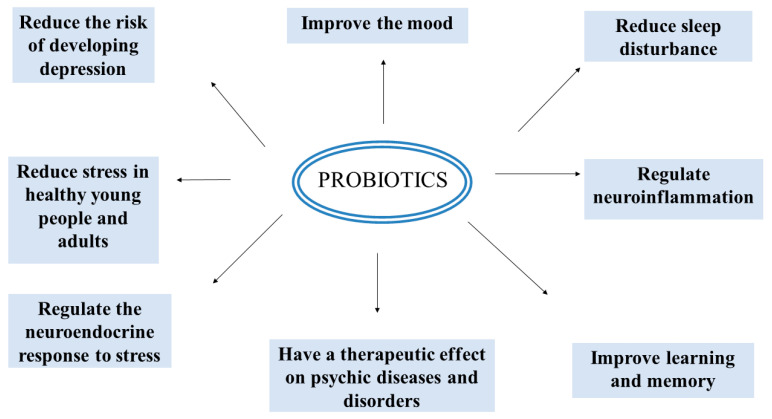
Some important effects of probiotics on mental well-being.

By way of conclusion, for future perspectives (or open questions) we propose the possible effects of psychobiotics and the trends for future research (Figure 2): regulation of neuroendocrine response to stress; improvement of memory and learning ability; mood improvement; and reduction in risk of depression, sleep disturbance, and stress, linked to a physiological effect on some inflammation markers.

## Figures and Tables

**Table 1:** Studies on probiotics found on ClinicalTrials.gov. The topic contains the link to the study.

Topic	Studies	Studies with Results
Vaginal Diseases	10	1
Liver Cirrhosis	10	1
Kidney Diseases	10	2
Fibrosis	10	1
Depressive Disorder	10	0
Depression	10	0
Cross Infection	10	2
Asthma	10	0
Vaginitis	11	1
Tooth Diseases	11	1
Metabolic Syndrome	11	1
HIV Infections	11	2
Anxiety Disorders	11	3
Acquired Immunodeficiency Syndrome	11	2
Diabetes Mellitus, Type 2	12	0
Brain Diseases	12	1
Abdominal Pain	12	0
Influenza, Human	12	0
Quality of Life	14	2
Pregnancy Complications	14	4
Lipid Metabolism Disorders	14	0
Infant, Newborn, Diseases	14	1
Behavioral Symptoms	14	1
Nutrition Disorders	15	0
Neurologic Manifestations	16	1
Endocrine System Diseases	16	0
Dyslipidemias	16	0
Central Nervous System Diseases	16	4
Diabetes Mellitus	17	0
Skin Diseases, Genetic	18	0
Respiratory Hypersensitivity	18	2
Psychotic Disorders	19	5
Mental Disorders	19	5
Liver Diseases	19	2
Skin Diseases, Eczematous	20	0
RNA Virus Infections	20	4
Nose Diseases	20	3
Eczema	20	0
Dermatitis	20	0
Otorhinolaryngologic Diseases	22	3
Insulin Resistance	22	1
Hyperinsulinism	22	1
Virus Diseases	24	4
Pain	24	1
Bacterial Infections	24	6
Skin Diseases	30	2
Glucose Metabolism Disorders	31	1
Obesity	36	1
Irritable Bowel Syndrome	39	0
Mouth Diseases	40	0
Constipation	42	3
Inflammation	43	3
Gastroenteritis	47	7
Stomatognathic Diseases	51	2
Respiratory Tract Infections	51	5
Respiratory Tract Diseases	59	6
Metabolic Diseases	61	2
Diarrhea	70	7
Intestinal Diseases	77	6

**Table 2:** Studies selected on ClinicalTrials.gov. The title of the study contains the link to the dashboard of the website.

Title of Studies	Probiotics	Results	Reference
Impact of Probiotics on Urinary Symptoms in Spinal Cord Injury SCI and SB *	*Lacticaseibacillus rhamnosus*, strain GG	Not clearly reported	Not available ***
Biological Signatures, Probiotic Among Those With mTBI and PTSD **	Dietary Supplement: of *Limosilactobacillus reuteri*	Probiotic supplementation resulted in a decrease in plasma C-reactive protein	[13]
Zinc and/or Probiotic Supplementation of Rotavirus and Oral Polio Virus Vaccines	*Lactobacillus rhamnosus*, strain GG	Not clearly reported	Not available ***
Double-Blind Trial of a Probiotic Supplement to Reduce the Symptoms of Schizophrenia	*Lacticaseibacillus rhamnosus*, strain GG, and *Bifidobacterium lanimalis*	Patients in the probiotic group were less likely to develop severe bowel difficulty over the course of the trial	[14]
Probiotics to Prevent Relapse After Hospitalization for Mania	*Lactocaseibacillus* strain GG and *Bifidobacterium animalis* subsp. *lactis* strain Bb12	Probiotic supplementation is associated with a lower rate of rehospitalization in patients who have been recently discharged following hospitalization for mania	[15]
Probiotics for Quality of Life in Autism Spectrum Disorders	Probiotic mix (VISBIOME)	Not clearly reported	Not available ***
Effect of Milk Oligosaccharides and Bifidobacteria on the Intestinal Microflora of Children with Autism	*Bifidobacterium infantis*	Not clearly reported	Not available ***
The Probiotic Study: Using Bacteria to Calm Your Mind	*Lacticaseibacillus rhamnosus*	The researchers reported the change in magnitude of self-reported states of anxiety, derived from summed raw scores obtained through the six-item short-form of the state scale of the Spielberger State-Trait Anxiety Inventory at baseline and 30 days from baseline.	[16]
Stress & Anxiety Dampening Effects of a Probiotic Supplement Compared to Placebo in Healthy Subjects	*Lacticaseibacillus paracasei* strain Lpc-37	Not clearly reported	Not available ***

* SCI, spinal cord injury; SB, spina bifida. ** TBI, traumatic brain injury; PTSD, post-traumatic stress disorder. *** The outputs/results of the study were not published; the record of this trial was found only on the database [11].

**Table 3:** Overview of possible psychobiotic effects of some species.

Probiotic or Mix	Effect
*Lactiplantibacillus plantarum*	Alleviation of cognitive symptoms in stressed adults. Improvement of cognitive performance.
*Lacticaseibacillus rhamnosus*	Reduction in anxiety and sleep disturbance. Reduction in the incidence of postpartum depression.
*Bifidobacterium breve*	Improvement in the HADS and PANSS score.
*Lacticaseibacillus rhamnosus* + *Bifidobacterium animalis*	Reduction in active antibodies against *C. albicans* (reduction in psychotic symptoms in schizophrenic patients).
*Lactobacillus helveticus + Bifidobacterium longum*	Improved BDI score.
*Bifidobacterium longum*	Reduction in cortisol production and stress in healthy adults.
Winclove’s Ecologic^®^ Barrier	Reduction in the risk of developing depression.

## Data Availability

Not applicable.

## Supplementary Materials

The following supporting information can be downloaded at: https://www.mdpi.com/article/10.3390/microorganisms10112141/s1, Table S1: Surveys and questionnaires for mental well-being [27,28,29,30,31,32,33,34,35,36,37,38,39,40,41,42,43,44,45,46,47,48,49,50,51,52,53,54,55].
