# Supplementary material for: Psycho-Microbiology, a New Frontier for Probiotics: An Exploratory Overview
Source: Microorganisms. 2022 Oct 29;10(11):2141. doi: 10.3390/microorganisms10112141 (PMC9696884; doi:10.3390/microorganisms10112141)
Supplement: Supplementary file 1 [file microorganisms-10-02141-s001.zip › microorganisms-1991202-supplementary.pdf]

Table S1: Surveys and questionnaires for mental well-being

**Anxiety Control Questionnaire-Revised (ACQ-R)**	The ACQ-R is a 15-item questionnaire that assesses an individual's perceived level of control over anxiety-related events. The ACQ-R produces a composite score (ACQ.Total) along with three subscales: emotion control (e.g., "I am able to control my level of anxiety"), threat control (e.g., "there is little I can do to change frightening events"), and stress control (e.g., "I usually find it hard to deal with difficult problems").
**Auditory Continuous Performance Test (ACPT)**	The ACPT measures selective and sustained attention by asking participants to respond by pressing a button as soon as they hear the target stimulus. Correct and incorrect detection, omission errors, and reaction time are measured while participants listen to random numbers for 9 min and respond to the target stimulus [27].
**Beck Anxiety Inventory (BAI)**	The BAI is a 21-item inventory that assesses the severity of anxiety level. The composite score (BAI.Total) ranges from 0 to 63 points (0–21 = low level of anxiety, 22–35 = moderate level of anxiety, and 36 or higher = high level of anxiety). The measure also produces four subscales that are used in the study: Neurophysiological anxiety (BAI.Neu) (e.g., "dizzy,"), Subjective anxiety (BAI.Sub) (e.g., "terrified"), Panic anxiety (BAI.Pan) (e.g., "difficulty breathing"), Autonomic anxiety (BAI.Aut) (e.g., "sweating") [28].
**Beck Depression Inventory (BDI)**	BDI is a self compiled questionnaire of 21 items in multiple-choice format [29]. Under each item, there are four statements, and the subjects were instructed to choose the one that best described their situation during the last 2 weeks. The declarations were given the scores 0, 1, 2, and 3, with 0 for the normal or least depressive statement and 3 for the most depressive statement. We calculated the total BDI score by adding together the scores of each item.
**Beck Depression Inventory – II (BDI-II)**	The BDI-II is a 21-item self-report measure of the presence and severity of depressive symptoms within the past two weeks [30]. The total score is interpreted via use of standardised cut-offs between 0 and 63 (0-13: minimal depression, 14-19: mild depression, 20-28: moderate depression and 29-63: severe depression) [31]. The BDI-II demonstrates good validity and reliability [30] and has been shown to be a valid diagnostic discriminator of depression [32].
**Cyberball Game (CBG)**	The CBG was used to study the effects of social stress and exclusion. Briefly, during the CBG, participants were asked to play a virtual ball-tossing game with 2 other virtual players programmed. The CBG involves 2 conditions, the so-called inclusion and exclusion [33]

**Depression Anxiety and Stress Scale-21 items (DASS-21)**	The DASS-21 is a clinical screening instrument with three separate subscales to measure levels of anxiety, depression and stress within the past week, and has been shown as a valid measure of symptoms in clinical settings [34,35]. The version used in this study is a 5 short, 21-question self-report version of the full 42 item questionnaire, with evidence of comparable validity and internal consistency to the full questionnaire [36].
**Depression, Anxiety and Stress Scale questionnaire (DASS-42)**	DASS-42 is a 42 item self-report validated inventory comprising of three scales designed to measure the negative emotional states of depression, anxiety and stress, where each of the three scales contained 14 items. The depression scale assessed dysphoria, hopelessness, devaluation of life, self-deprecation, lack of interest/involvement, anhedonia, and inertia. The anxiety scale assessed autonomic arousal, skeletal muscle effects, situational anxiety, and subjective experience of anxious affect, while the stress scale assessed difficulty in relaxing, nervous arousal, and being easily upset/agitated, irritable/over-reactive and impatience. Subjects are assessed based on a 4- point Likert scale (0 = did not apply to me at all, 1 = applied to me to some degree or some of the time, 2 = applied to me to a considerable degree or a good part of time, 3 = applied to me very much or most of the time). Scores for each subscale were categorized into five severity ranges, namely normal, mild, moderate, severe and extremely severe [34].
**Digit Span Test (DST)**	The DST was used to evaluate working memory by asking participants to immediately recall a sequence of numbers. Outcome measures of list memory and tracking scores indicate the number of correct responses and the longest digit span length correctly recalled, respectively [37].
**Edinburgh Postnatal Depression Scale (EPDS)**	The EPDS is a 10 item screening questionnaire widely used to assess maternal mood [38].
**Hospital Anxiety And Depression Scale (HADS)**	The HADS consists of 14 items scored on a 4-point Likert scale ranging from 0 to 3. The total score (0–42) reflects the severity of depressive and anxiety symptoms, with higher scores indicating more severe symptoms [39,40].

**Leiden Index of Depression Sensitivity-Revised (LEIDS-R)**	Leiden Index of Depression Sensitivity – Revised (LEIDS-R): The LEIDS-R is a 34-item self-report measure of cognitive reactivity to sad mood, which measures the extent to which dysfunctional thinking patterns are activated during periods of low mood [41]. Example items include "When I feel down, I more often feel hopeless about everything" and "When I feel down, I take fewer risks", and overall the LEIDS-R has shown to have good validity [42]. High cognitive reactivity has been to predict episodes of depression and is associated with a history of depressive episodes [43,44].
**Mini-International Neuropsychiatric Interview (M.I.N.I.)**	The M.I.N.I is a brief structured clinical interview based on the Diagnostic and Statistical Manual – 4th Edition (DSM-IV) and the International Statistical Classification of Diseases and Related 4 Health Problems – 10th Edition (ICD-10). The module on Major Depressive Episode and Major Depressive Disorder was administered in this clinical trial, and was a 14-item clinician-led structured interview which assessed for current and previous episodes of depression. The M.I.N.I has been found to possess good validity and reliability as assessed against the Structured Clinical Interview for DSM-III-R-Patients [45], and has good concurrent validity with psychiatrist evaluations of individuals with depressive symptoms [46]. It can be administered within a shorter time frame, making it a suitable choice for this study.
**Mood Questionnaire (MQ)**	The MQ is used to assess mood, using 8 questions (are you feeling bad, good, happy, sad, pleasant, angry, friendly, and unfriendly), all rated between 1 and 5.
**Negative Mood Regulation (NMR)**	The NMR is a 30-item questionnaire assessing an individual's perception of their ability to regulate negative mood (e.g., "wallowing in it is all I can do"). The composite score (NMR.Total) ranges from 30 to 150 (higher scores = higher control of negative mood). The scale has good internal consistency (α ranges from 0.86 to 0.92); good temporal stability, with test–retest correlations ranging from 0.73 to 0.74 for 4 weeks and from 0.67 to 0.78 for a 6 to 8-week interval; a unidimensional factor structure; consistent relations with self-reported depression; and discriminant validity from social desirability and locus of control [47].

**Need Threat Scale (NTS)**	The NTS is designed to measure the feelings and emotional consequences of social rejection, and higher scores related to higher distress level. Its 4 items (rated between 1 and 5 for "weak" to "strong") comprised self-esteem, belonging, meaningful existence, and control, and combined ratings have been used as a measure of social distress in previous studies
**Penn State Worry Questionnaire (PSWQ)**	The PSWQ is a 16-item questionnaire that measures trait worry (e.g., "many situations make me worry"). The composite score (PSWQ.Total) can be interpreted as follows: 16–39 = low level of worry, 40–59 = moderate level of worry, and 60–80 = high level of worry. The coefficient alpha for the final PSWQ was 0.93. It was found that the PSWQ had good test–retest reliability and validity [48].
**Perceived Stress Scale questionnaire (PSS-10)**	PSS-10 consisted of ten-items on a 5-point Likert scale (0 = never, 1 = almost never, 2 = sometimes, 3 = fairly often, 4 = very often), where six items were negatively stated while four items that were positively stated (items 4, 5, 7, and 8) were reversely scored (0 = very often, 1 = fairy often, 2 = sometimes, 3 = almost never, 4 = never). The sum of the 10 items represented the total score, with scores of 0-13 indicating low stress, 14-26 indicating moderate stress and 27-40 indicating high perceived stress [49].
**Pittsburgh Sleep Quality Index (PSQI)**	The PSQI is a self-rated questionnaire which assesses sleep quality and disturbances over a 1-month time interval [50].
**Positive And Negative Affect Schedule (PANAS)**	The PANAS questionnaire consisted of 20 items measuring positive affect (10-items) and negative affect (10-items). For positive affect (PANAS.Pos) (e.g., "excited"), high scores represent high positive affect, while high scores on negative affect (PANAS.Neg) (e.g., "ashamed,") represent high negative affect [51].
**Positive and Negative Syndrome Scale (PANSS)**	The PANSS [52] consists of 30 items scored on a 7-point Likert scale ranging from 1 to 7, with higher scores indicating more severe symptoms. Based on a five-factor model of the PANSS [53], the sum of the anxiety and depressive symptoms (items 1, 2, 3, 4, and 6 of the general psychopathology subscale) ranging from 5 to 35 gives the PANSS anxiety and depressive symptoms score, which reflects the severity of anxiety and depressive symptoms in patients with schizophrenia.
**Spielberger State-Trait Anxiety Inventory (STAI)**	The STAI is a commonly used measure of trait and state anxiety. It can be used in clinical settings to diagnose anxiety and to distinguish it from depressive syndromes. It also is often used in research as an indicator of caregiver distress [54]

**Subjective Exclusion Perception (SEP)**	The SEP is used to record participants' feeling of being included/ostracized by asking them to rate 2 statements ("I was ignored" and "I was excluded") between 1 and 5.
**State Trait Anxiety Inventory 6 item version (STAI6)**	The STAI6 is a short 6 item scale validated as an anxiety screening questionnaire based on the longer State Trait Anxiety Inventory [16].
**Verbal Learning Test (VLT)**	For the VLT, 15 words are read aloud by an examiner and the participants are required to memorize the list. Participants receive scores for immediate recall, delayed recall, and recognition to evaluate verbal memory function.
**28-Item General Health Questionnaire (GHQ-28)**	The General Health Questionnaire is self-report screening measure used to detect possible psychological disorder. The GHQ-28 identifies two main concerns: the inability to carry out normal functions; and the appearance of new and distressing phenomena [55].
